# Negative pressure wound therapy: Potential publication bias caused by lack of access to unpublished study results data

**DOI:** 10.1186/1471-2288-8-4

**Published:** 2008-02-11

**Authors:** Frank Peinemann, Natalie McGauran, Stefan Sauerland, Stefan Lange

**Affiliations:** 1 Institute for Quality and Efficiency in Health Care (IQWiG), Dillenburger Str. 27, 51105 Cologne, Germany; 2 Institute for Research in Operative Medicine, University of Witten/Herdecke, Ostmerheimer Str. 200, 51109 Cologne, Germany

## Abstract

**Background:**

Negative pressure wound therapy (NPWT) is widely applied, although the evidence base is weak. Previous reviews on medical interventions have shown that conclusions based on published data alone may no longer hold after consideration of unpublished data. The main objective of this study was to identify unpublished randomised controlled trials (RCTs) on NPWT within the framework of a systematic review.

**Methods:**

RCTs comparing NPWT with conventional wound therapy were identified using MEDLINE, EMBASE, CINAHL and The Cochrane Library. Every database was searched from inception to May 2005. The search was updated in December 2006. Reference lists of original articles and systematic reviews, as well as congress proceedings and online trial registers, were screened for clues to unpublished RCTs. Manufacturers of NPWT devices and authors of conference abstracts were contacted and asked to provide study information. Trials were considered nonrandomised if concealment of allocation to treatment groups was classified as "inadequate". The study status was classified as "completed", "discontinued", "ongoing" or "unclear". The publication status of completed or discontinued RCTs was classified as "published" if a full-text paper on final study results (completed trials) or interim results (discontinued trials) was available, and "unpublished" if this was not the case. The type of sponsorship was also noted for all trials.

**Results:**

A total of 28 RCTs referring to at least 2755 planned or analysed patients met the inclusion criteria: 13 RCTs had been completed, 6 had been discontinued, 6 were ongoing, and the status of 3 RCTs was unclear. Full-text papers were available on 30% of patients in the 19 completed or discontinued RCTs (495 analysed patients in 10 published RCTs vs. 1154 planned patients in 9 unpublished RCTs). Most information about conference abstracts and unpublished study information referring to trials that were unpublished at the time these documents were generated was obtained from the manufacturer Kinetic Concepts Inc. (KCI) (19 RCTs), followed by The Cochrane Library (18) and a systematic review (15). We were able to obtain some information on the methods of unpublished RCTs, but results data were either not available or requests for results data were not answered; the results of unpublished RCTs could therefore not be considered in the review. One manufacturer, KCI, sponsored the majority of RCTs (19/28; 68%). The sponsorship of the remaining trials was unclear.

**Conclusion:**

Multi-source comprehensive searches identify unpublished RCTs. However, lack of access to unpublished study results data raises doubts about the completeness of the evidence base on NPWT.

## Background

Conservative estimates report that about 1% of the population in Western countries is affected by chronic wounds [1], with a much higher prevalence rate in inpatient and nursing facilities [2]. Besides presenting a significant risk factor for complications such as infection and amputation, chronic wounds also lead to marked impairment of patients' quality of life [3,4].

Negative pressure wound therapy (NPWT) consists of an open-cell foam dressing covered with an adhesive drape. The dressing is connected to a vacuum pump that creates and maintains a subatmospheric pressure [5]. The most commonly used NPWT device is the Vacuum Assisted Closure (V.A.C.^®^) device.

The Federal Joint Committee (*Gemeinsame Bundesausschuss*), the decision-making body of the self-administration of the German health care service, commissioned the Institute for Quality and Efficiency in Health Care (*Institut für Qualität und Wirtschaftlichkeit im Gesundheitswesen, IQWiG*) to conduct a systematic review on the efficacy and safety of NPWT versus conventional wound therapy in patients with acute or chronic wounds. The full review was published in German in March 2006, and an English-language article has recently been published [6,7]. An update of the systematic review, a rapid report, was published in German in January 2007 [8].

Although NPWT is widely applied, particularly for chronic wounds, at the time of the review only a few relevant randomised controlled trials (RCTs) were available; these were, moreover, of poor quality [9]. Previous reviews on NPWT have not provided clear evidence of the superiority of NPWT over conventional wound therapy. For example, a review published by the Canadian Medical Advisory Secretariat in 2006 concluded that "Based on the evidence to date, the clinical effectiveness of NPWT to heal wounds is unclear" [10].

Previous reviews on medical interventions have shown that conclusions on efficacy and safety based on published data alone may no longer hold after consideration of unpublished data, which may reverse favourable risk-benefit profiles and attenuate treatment effects of an apparently superior intervention [11-13]. Within the framework of the systematic review on NPWT, our main objective was therefore not only to identify published, but also unpublished completed or discontinued RCTs in order to gain as complete an overview as possible of the evidence on NPWT. Further aims were to locate ongoing RCTs for potential consideration in future updates, and to determine the type of sponsorship of all eligible trials.

## Methods

The literature search comprised a total of 7 steps. Table 1 shows an overview of the sources used.

**Table 1:** Literature sources and search steps*

**Category**	**Sources**
**Step 1**	
Bibliographic databases^†^	MEDLINE: Includes PubMed and Clinical Queries. EMBASE The Cochrane Library. These databases were searched: Cochrane Database of Systematic Reviews (CDSR; Cochrane Reviews) Database of Abstracts of Reviews of Effects (DARE; Other Reviews) Cochrane Central Register of Controlled Trials (CENTRAL; Clinical Trials) Health Technology Assessment Database (HTA; Technology Assessments) NHS Economic Evaluation Database (NHSEED; Economic Evaluations) CINAHL

**Step 2**	
Online trial registers	ClinicalTrials.gov National Research Register (NRR)

**Step 3**	
Authorities	German Federal Joint Committee (references cited by experts in 2003) United States Federal Drug Administration (FDA) (online search) German Federal Institute for Drugs and Medical Devices (direct enquiry by e-mail and phone)

**Step 4**	
Systematic reviews^‡^	Ontario Health Technology Advisory Committee 2006 (OHTAC) Pham 2006, Australian Safety and Efficacy Register of New Interventional Procedures – Surgical (ASERNIP-S) Costa 2005, McGill University Health Centre Technology Assessment Unit (MUHC) Samson 2004, United States Agency for Healthcare Research and Quality (AHRQ) Higgins 2003, Centre for Clinical Effectiveness (CCE) Fisher 2003, Canadian Coordinating Office of Health Technology Assessment (CCOHTA) Evans 2001, Cochrane database of systematic reviews (CDSR)

**Step 5**	
Congress proceedings	V.A.C.^® ^Wundtherapie, 10 Jahre V.A.C., Drei-Länder-Kongress [V.A.C. Wound Therapy, 10 Years of V.A.C., Three-Country Conference], Graz, Austria; 2005. Symposium on Advanced Wound Care, San Diego, California, USA; 2005. Second World Union of Wound Healing Societies Meeting, Paris, France; 2004. V.A.C.^® ^Wundtherapie, Drei-Länder-Kongress [V.A.C. Wound Therapy, Three-Country Conference], Mainz, Germany; 2004.
	First international topical negative pressure (TNP) therapy focus group meeting in London, UK, December 2003. Proceedings. Edited by: Banwell P, Teot L. Supported by the European Tissue Repair Society. Faringdon, UK: TXP Communications; 2004. Vacuum Assisted Closure (V.A.C.^®^), Salzburg, Austria; 2003. Eleventh Annual Meeting and Educational Symposium, Wound Healing Society, Albuquerque, New Mexico, USA; 2001.

**Step 6**	
Manufacturers (direct enquiries by e-mail and post)	Kinetic Concepts, Inc., San Antonio, Texas, USA. BlueSky Medical Group, Inc., Carlsbad, California, USA

**Step 7**	
Authors (direct enquiries by e-mail and post)	Adams; Armstrong; Bayer; Foo; Fryer; Greer; Gupta; Heath; Lantis; McCarthy J; McCarthy M; Molnar; Niezgoda; Orgill; Payne; Stannard (3 RCTs); Vuerstaek; Walker

* Other sources available on request.

† Search executed in May 2005 and updated in December 2006.

‡ Hayes 2003 [71] was not publicly available and therefore not considered. OHTAC 2004 [72] and Pham 2003 [73] were not considered because updated versions are available.

We searched 4 bibliographic databases (MEDLINE, EMBASE, CINAHL, and The Cochrane Library) for RCTs on NPWT versus conventional wound therapy in patients with acute or chronic wounds. All databases were searched from inception to May 2005. The search was updated in December 2006 within the framework of the preparation of the rapid report on NPWT [8]. The search strategy in MEDLINE (Table 2) was applied according to the Cochrane Highly Sensitive Search Strategy described in the Cochrane Handbook for Systematic Reviews of Interventions [14], and tailored to the requirements of each database. Other search strategies are available upon request.

**Table 2:** Search strategy applied in MEDLINE

**No.**	**Search terms**
1	amputation$.ti,ab.
2	exp AMPUTATION/
3	exp AMPUTATION TRAUMATIC/
4	burn$.ti,ab.
5	exp BURNS/
6	decubit$.ti,ab.
7	deglov$.ti,ab.
8	diabet$.ti,ab.
9	exp DIABETES MELLITUS/
10	electric$ injur$.ti,ab.
11	frostbit$.tw.
12	exp FROSTBITE/
13	laceration$.ti,ab.
14	exp LACERATIONS/
15	open-abdom$.ti,ab.
16	exp ABDOMINAL WALL/su
17	plastic-surg$.ti,ab.
18	exp SURGERY, PLASTIC/
19	reconstruct$-surg$.ti,ab.
20	exp RECONSTRUCTIVE SURGICAL PROCEDURES/
21	skin-graft$.ti,ab.
22	skin-transplant$.ti,ab.
23	exp SKIN TRANSPLANTATION/
24	surg$ flap.ti,ab.
25	exp SURGICAL FLAPS/
26	thermal injur$.ti,ab.
27	exp ELECTRIC INJURIES/
28	ulcer$.ti,ab.
29	ul#us$.ti,ab.
30	exp SKIN ULCER/
31	exp SOFT TISSUE INFECTIONS/
32	exp ULCER/
33	wound$.ti,ab.
34	exp WOUND INFECTION/
35	exp WOUND HEALING/
36	wound dehiscence.ti,ab.
37	exp SURGICAL WOUND DEHISCENCE/
38	"mini-v.a.c.$".ti,ab.
39	negative-pressur$.ti,ab.
40	subatmospheric-pressur$.ti,ab.
41	sub-atmospheric-pressur$.ti,ab.
42	$suction$.ti,ab.
43	exp SUCTION/
44	vacuum$.ti,ab.
45	exp VACUUM/
46	(clin$ adj25 trial$).ti,ab.
47	clinical trial.pt.
48	exp CLINICAL TRIALS/
49	(control$ or prospectiv$ or volunteer$).ti,ab.
50	controlled clinical trial.pt.
51	COMPARATIVE STUDY.sh.
52	DOUBLE BLIND METHOD.sh.
53	exp EVALUATION STUDIES/
54	FOLLOW-UP STUDIES.sh.
55	placebo$.ti,ab.
56	PLACEBOS.sh.
57	PROSPECTIVE STUDIES.sh.
58	random$.ti,ab.
59	randomized controlled trial.pt.
60	RANDOM ALLOCATION.sh.
61	RANDOMIZED CONTROLLED TRIALS.sh.
62	RESEARCH DESIGN.sh.
63	SINGLE BLIND METHOD.sh.
64	((singl$ or doubl$ or trebl$ or tripl$) adj25 (blind$ or mask$)).ti,ab.
65	or/1–37
66	or/38–45
67	or/46–65
68	and/66–68

Note: We have amended or deleted 2 terms contained in the original strategy. The first term "frostbite$.ti,ab." was replaced by "frostbit$.tw.". The second term "(metaanaly$ or (meta and analy$) or ((review or search$) and (systemat$))).ti,ab." was deleted. The amended strategy did not affect the search results. However, as an article may be published in the future and missed by not truncating the first term at the appropriate spot, the term "frostbit$.tw." should be used. The second term is superfluous.

We then screened online trial registers (Table 1) and the US Food and Drug Administration website, and contacted German authorities, requesting information on all available RCTs on NPWT. Prior to the start of the review, the Federal Joint Committee had asked experts to provide relevant literature; these citations were also obtained by IQWiG as part of the commission. Moreover, the reference lists of retrieved original articles and systematic reviews, as well as congress proceedings, were handsearched.

Two manufacturers of NPWT devices were identified: Kinetic Concepts Inc. (KCI, San Antonio, Texas, USA) markets the V.A.C.^® ^device, and BlueSky Medical Group Inc. (BSM, Carlsbad, California, USA) markets the Versatile 1 Wound Vacuum System^®^. Both were asked to provide information on the study status and publication status of sponsored trials, as well as on methods and results. Finally, we contacted the authors of conference abstracts by e-mail and by post. All contact details of the relevant institutions were checked beforehand on the corresponding websites. Manufacturers and authors were informed that IQWiG does not accept "commercial in confidence" data and publishes all data contributing to a systematic review [15].

All languages were included, as long as a title was available in English. If the title indicated a potential relevance of the study, the corresponding article was obtained and translated. Only RCTs comparing NPWT versus conventional therapy were eligible. Trials were considered nonrandomised if concealment of allocation to treatment groups was classified as "inadequate" [16]. The intervention was classified as NPWT if a medical device system identical or comparable to the V.A.C.^® ^Therapy System was used.

Results of trials that were not available as full-text publications were only considered in the review if detailed data were available (e.g. from a clinical study report or a manuscript in press). Results data from conference abstracts were not considered.

All steps of the literature screening were performed by two reviewers independently of one another. Any disagreements were resolved by discussion. The titles and abstracts of the retrieved documents were screened to exclude citations that were clearly irrelevant. The full texts of the remaining potentially relevant articles were then screened to identify RCTs that fulfilled the inclusion criteria stated above.

With the help of the information obtained, the study status of the RCTs identified was classified as "completed", "discontinued", "ongoing" or "unclear". The terminology used in the literature to classify unpublished data or so-called "grey literature" is inconsistent [12]. We classified the publication status of completed or discontinued trials as "published" if a full-text paper on final study results (completed trials) or interim results (discontinued trials) was identified in the literature search, and "unpublished" if this was not the case. In addition, for ongoing trials it was indicated whether interim results were available or not. The type of sponsorship was also noted for all trials.

Only a summary of results on the quality assessment and outcomes of the RCTs included in the IQWiG systematic review and rapid report is presented here, as the main focus of this paper was to identify unpublished RCTs.

## Results

An overview of the search results is presented in Table 3. Detailed information on identified published and unpublished RCTs, as well as on the RCTs included in the systematic review and rapid report, is presented in Table 4.

**Table 3:** Overview of study status, publication status, and sponsorship (Status: December 2006)

	**Study status**				
		
	**Completed **(trials/patients)	**Discontinued **(trials/patients)	**Ongoing **(trials/patients)	**Unclear **(trials/patients)	**Total **(trials/patients)
**Publication status**					
Published*	9/473	1/22	(2/88)	(0/0)	10/495^†^
Unpublished^‡^	4/328	5/826	(4/500)	(3/?)	9/1154^†^

**Sponsorship**					
Industrial	8/413	6/848	5/1076	0/0	19/2337
Unclear	5/388	0/0	1/30	3/?	9/418

**Total**	13/801	6/848	6/1106	3/?	28/2755

* Analysed patients.

† Published/unpublished: full-text publications of final results (completed trials) or interim results (discontinued trials) were available/not available.

‡ Planned patients.

**Table 4:** Search results for randomised controlled trials on negative pressure wound therapy* (Status: December 2006)

**Study status**	**No.**	**Abstract**	**Information source for abstract**	**Sponsor (ID^†^)**	**Reply to inquiry to sponsor or author**	**Patients planned^‡^**	**Patients analysed^§^**	**Results data available**	**Full-text publication available^||^**
**Completed**									
	1	Vuerstaek [74]	WUWHS 2004	KCI (VAC VLU)	KCI	-	60	Yes^¶^	Vuerstaek 2006 [23]
	2	No abstract	-	Unclear	-	-	60	Yes^¶^	Llanos 2006 [25]
	3	Obdeijn [75]	WUWHS 2004	KCI (No ID)	KCI	-	65	Yes^¶^	Braakenburg 2006 [21]
	4	Payne [76]	WUWHS 2004	KCI (VAC 2001–07)	KCI; author	-	162	Yes^#^	Armstrong 2005 [22]
	5	Moues [77]	WUWHS 2004	KCI (PMID 14974959)	KCI	-	54	Yes^#^	Moues 2004 [20]
	6	Heath [78]	The Cochrane Library	KCI (PMID 15468399)	KCI; author	-	20	Yes^#^	Moisidis 2004 [18]
	7	No abstract	-	KCI (PMID 14534844)	KCI	-	6	Yes^#^	Eginton 2003 [17]
	8	No abstract	-	KCI (PMID 12625392)	KCI	-	22	Yes^#^	Wanner 2003 [24]
	9	Joseph [79]	The Cochrane Library	KCI (No ID)	KCI	-	24	Yes^#^	Joseph 2000 [19]
	10	Adams [26]	NRR	Unclear	No reply	Unclear	Unclear	No	No
	11	Fryer [27]	ClinicalTrials.gov	Unclear	No reply	120	Unclear	No	No
	12	McCarthy [28]	NRR	Unclear	Author **	160	Unclear	No	No
	13	Walker [29]	NRR	Unclear	No reply	48	Unclear	No	No
***Subtotal***	13					328	473		

**Discontinued**									
	14	No abstract	-	KCI (PMID 12142596)	KCI	-	22	Yes^#^	Interim results: Ford 2002 [30]
	15	No abstract	KCI	KCI (VAC 2001–02)	KCI	214	-	No	No
	16	Bayer [31]	WUWHS 2004	KCI (VAC 2002–09)	KCI	116	-	No	No
	17	Greer [33]	The Cochrane Library	KCI (No ID)	KCI	^†† ^80	-	No	No
	18	Orgill [34]	WUWHS 2004	KCI (VAC 2002–10)	KCI	116	-	No	No
	19	Stannard [35]	WUWHS 2004	KCI (VAC 2001–06)	KCI; author	300	-	No	No
***Subtotal***	6					826	22		

**Ongoing**									
	20	Armstrong [36]	WUWHS 2004	KCI (VAC 2001–08)	KCI; author	206	-	-	No
	21	McCarthy [37]	ClinicalTrials.gov	Unclear	No reply	30	-	-	No
	22	Molnar [38]	WUWHS 2004	KCI (VAC 2001–00)	KCI	50	-	-	No
	23	Niezgoda [39]	WUWHS 2004	KCI (VAC 2001–01)	KCI	214	-	-	No
	24	Stannard [40]	WUWHS 2004	KCI (VAC 2001–04)	KCI; author	258	(44)	Yes^¶^	Interim results: Stannard 2006 [80]
	25	Stannard [41]	WUWHS 2004	KCI (VAC 2001–05)	KCI; author	348	(44)	Yes^¶^	Interim results: Stannard 2006 [80]
***Subtotal***	6					1106	(88)		

**Unclear**									
	26	Foo [42]	WUWHS 2004	Unclear	No reply	Unclear	-	No	No
	27	Gupta [43]	WHS 2001	Unclear	No reply	Unclear	-	No	No
	28	Lantis [44]	WUWHS 2004	Unclear	No reply	Unclear	-	No	No
***Subtotal***	3					Unclear			

**Total (all trials)**	28					2260	495		

* Not classified by IQWiG as an RCT on NPWT (in contrast to other systematic reviews): McCallon 2000 [69]; Genecov 1998 [70]; Buttenschoen 2001 [81]; Jeschke 2004 [82].

† The PubMed ID number, when available, is used by KCI as the study ID for published RCTs.

‡ Number of patients planned in unpublished and ongoing studies as stated in abstracts and personal communications.

§ Number of patients analysed in published studies.

|| Full-text publication of interim or final results (any study status).

¶ Results considered in the IQWiG rapid report.

# Results considered in the IQWiG systematic review.

** The author replied to IQWiG's first request for information and stated that the trial was ongoing. However, according to information provided by the NRR (accessed on 04 Feb 2007), this trial has since been completed. A second query was not answered.

†† According to KCI, 16 patients were enrolled in this trial.

ID: Identification code; NRR: National Research Register; PMID: PubMed Identification Code; VLU: venous leg ulcer; WHS: Wound Healing Society; WUWHS: World Union of Wound Healing Societies.

### Search results

**Figure 1:**
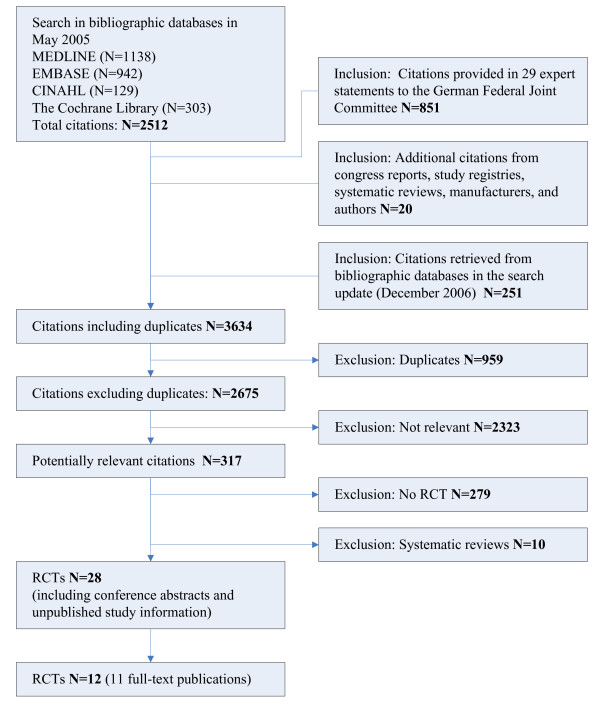
Flow chart of the literature search for RCTs.

Of 2675 potentially relevant publications, 317 full-text papers were obtained for further assessment. Of these, 289 were excluded as not relevant (Figure 1).

A total of 28 RCTs on NPWT referring to at least 2755 planned or analysed patients were identified: 13 RCTs (801 patients; 29%) had been completed [17-29], 6 RCTs (848 patients; 31%) had been discontinued [30-35], 6 RCTs (1106 patients; 40%) were ongoing [36-41], and the status of 3 RCTs was unclear [42-44] (Table 3). According to KCI, the main reasons for discontinuation were slow enrolment, high attrition rates, changes in clinical practice, or design flaws [6].

Full-text publications were available on 30% of the 1649 patients in the 19 completed or discontinued RCTs (495 analysed patients in 10 published RCTs vs. 1154 planned patients in 9 unpublished RCTs). Of the 14 conference abstracts on these 19 trials, 6 abstracts (43%) were later published as full-text articles.

### Response behaviour of manufacturers and authors

A total of 17 authors and 2 manufacturers were contacted, of whom 10 (59%) and 2 (100%) responded respectively. KCI readily provided information on study and publication status and on methodological issues. BSM did not sponsor relevant RCTs and provided information only on case reports.

One author provided information on an RCT that had been classified as "ongoing", and stated that this trial had recently been completed and the manuscript submitted to *The Lancet *[22]. As this trial included 162 analysed patients and was the largest RCT on NPWT conducted so far, IQWiG postponed the publication of its review by 2 months until the final results of this RCT were available, in order to include the results in the body of evidence.

### Literature sources

The sources of the conference abstracts and unpublished study information referring to 28 RCTs on NPWT are shown in Tables 5, 6: most information was obtained from KCI (19), followed by Cochrane (18), a systematic review (15) [9], and congress proceedings (14) [45].

**Table 5:** Sources of conference abstracts and unpublished study information on 28 RCTs: databases, trial registers, and systematic reviews

**No**	**Abstracts or other sources**	**Bibliographic databases**		**Trial registers**		**Systematic reviews**			
		
		**MEDLINE, EMBASE or CINAHL**	**Cochrane Clinical Trials**	**CT.gov**	**NRR**	**Evans 2001 [83]**	**Samson 2004 [9]**	**Pham 2006 [68]**	**Fisher 2003 [84]; Higgins 2003 [85]; Costa 2005 [67]; OHTAC 2006 [10]**
1	Vuerstaek [74]	-	+	-	-	-	+	-	-
2	No abstract*	-	-	-	-	-	-	-	-
3	Obdeijn [75]	-	+	-	-	-	+	-	-
4	Payne [76]	-	+	-	-	-	+	-	-
5	Moues [77]	-	+	-	-	-	+	-	-
6	Heath [78]	-	+	-	-	+	-	+	-
7	No abstract^†^	-	-	-	-	-	-	-	-
8	No abstract^‡^	-	-	-	-	-	-	-	-
9	Joseph [79]	-	+	-	-	-	-	-	-
10	Adams [26]	-	-	-	+	-	-	+	-
11	Fryer [27]	-	-	+	-	-	-	+	-
12	McCarthy [28]	-	-	-	+	-	-	+	-
13	Walker [29]	-	-	-	+	-	-	+	-
14	No abstract^§^	-	-	-	-	-	-	-	-
15	No abstract^||^	-	-	-	-	-	+	-	-
16	Bayer [31]	-	+	-	-	-	+	-	-
17	Greer [33]	-	+	-	-	+	-	+	-
18	Orgill [34]	-	+	-	-	-	+	-	-
19	Stannard [35]	-	+	-	-	-	+	-	-
20	Armstrong [36]	-	+	-	-	-	+	-	-
21	McCarthy [37]	-	-	+	-	-	-	-	-
22	Molnar [38]	-	+	-	-	-	+	-	-
23	Niezgoda [39]	-	+	-	-	-	+	-	-
24	Stannard [40]	-	+	-	-	-	+	-	-
25	Stannard [41]	-	+	-	-	-	+	-	-
26	Foo [42]	-	+	-	-	-	+	-	-
27	Gupta [43]	-	+	-	-	-	-	-	-
28	Lantis [44]	-	+	-	-	-	+	-	-

		**0**	**18**	**2**	**3**	**2**	**15**	**6**	**0**

* No preceding abstract was identified concerning the full-text article by Llanos 2006 (Tables 7–9).

† No preceding abstract was identified, but information by the manufacturer KCI was provided concerning the full-text article by Eginton 2003 (Tables 7–9).

‡ No preceding abstract was identified, but information by the manufacturer KCI was provided concerning the full-text article by Wanner 2003 (Tables 7–9).

§No preceding abstract was identified, but information by the manufacturer KCI was provided concerning the full-text article by Ford 2002 (Tables 7–9).

|| No preceding abstract was identified, but information by the manufacturer KCI was provided concerning this discontinued study KCI ID VAC 2001–02.

CT.gov: ClinicalTrials.gov (United States); NRR: National Research Register (United Kingdom National Health System); RCT: randomised controlled trial

**Table 6:** Sources of conference abstracts and unpublished study information on 28 RCTs: manufacturers, authors, congress proceedings, and authorities

**No**	**Abstracts or other sources**	**Manufacturers**		Authors*	Congress proceedings*		Authorities*	
				
		**Kinetic Concepts, Inc.**	**Blue Sky Medical**		**WUWHS 2004 [45]**	**Others**	**Experts 2003^†^**	**Others**
1	Vuerstaek [74]	+	-	-	+	-	-	-
2	No abstract^‡^	-	-	-	-	-	-	-
3	Obdeijn [75]	+	-	-	+	-	+	-
4	Payne [76]	+	-	+	+	-	-	-
5	Moues [77]	+	-	-	+	-	+	-
6	Heath [78]	+	-	+	-	-	-	-
7	No abstract^§^	+	-	-	-	-	-	-
8	No abstract^||^	+	-	-	-	-	-	-
9	Joseph [79]	+	-	-	-	-	+	-
10	Adams [26]	-	-	-	-	-	-	-
11	Fryer [27]	-	-	-	-	-	-	-
12	McCarthy [28]	-	-	+	-	-	-	-
13	Walker [29]	-	-	-	-	-	-	-
14	No abstract^¶^	+	-	-	-	-	-	-
15	No abstract^#^	+	-	-	-	-	-	-
16	Bayer [31]	+	-	-	+	-	-	-
17	Greer [33]	+	-	-	-	-	+	-
18	Orgill [34]	+	-	-	+	-	-	-
19	Stannard [35]	+	-	-	+	-	-	-
20	Armstrong [36]	+	-	+	+	-	-	-
21	McCarthy [37]	-	-	-	-	-	-	-
22	Molnar [38]	+	-	-	+	-	-	-
23	Niezgoda [39]	+	-	-	+	+	-	-
24	Stannard [40]	+	-	-	+	-	-	-
25	Stannard [41]	+	-	-	+	-	-	-
26	Foo [42]	-	-	-	+	-	-	-
27	Gupta [43]	-	-	-	-	+	-	-
28	Lantis [44]	-	-	-	+	-	+	-

		**19**	**0**	**4**	**14**	**2**	**5**	**0**

* See Table 1.

† Citations submitted by experts to the German Federal Joint Committee.

‡ No preceding abstract was identified concerning the full-text article by Llanos 2006 (Tables 7–9).

§No preceding abstract was identified, but information by the manufacturer KCI was provided concerning the full-text article by Eginton 2003 (Tables 7–9).

|| No preceding abstract was identified, but information by the manufacturer KCI was provided concerning the full-text article by Wanner 2003 (Tables 7–9).

¶No preceding abstract was identified, but information by the manufacturer KCI was provided concerning the full-text article by Ford 2002 (Tables 7–9).

# No preceding abstract was identified, but information by the manufacturer KCI was provided concerning this discontinued study KCI ID VAC 2001–02.

RCT: randomised controlled trial; WUWHS: World Union of Wound Healing Societies

The sources of all 11 published full-text articles (referring to 12 completed, discontinued or ongoing RCTs) are shown in Tables 7, 8, 9. All 11 articles were obtained from the Cochrane Library followed by KCI (10), MEDLINE (10), and EMBASE (9).

**Table 7:** Sources of 11 published full-text articles on 12 RCTs: databases and trial registers

**No**	**Full-text articles**	**Bibliographic databases**				**Trial registers**	
		
		**MEDLINE**	**EMBASE**	**Cochrane Clinical Trials**	**CINAHL**	**CT.gov**	**NRR**
1	Braakenburg 2006 [21]	+	+	+	-	-	-
2	Stannard 2006 [80]*	+	+	+	-	-	-
3	Vuerstaek 2006 [23]	+	-	+	-	+	-
4	Llanos 2006 [25]	+	+	+	-	-	-
5	Armstrong 2005 [22]	+	+	+	+	+	-
6	Moisidis 2004 [18]	+	+	+	-	-	-
7	Moues 2004 [20]	+	+	+	+	-	-
8	Eginton 2003 [17]	+	+	+	-	-	-
9	Wanner 2003 [24]	+	+	+	-	-	-
10	Ford 2002 [30]	+	+	+	-	-	-
11	Joseph 2000 [19]	-	-	+	+	-	-

		**10**	**9**	**11**	**3**	**2**	**0**

* Stannard 2006 [80]: one article presenting preliminary results of 2 ongoing RCTs [40,41].

CT.gov: ClinicalTrials.gov (United States); NRR: National Research Register (United Kingdom National Health System); RCT: randomised controlled trial

**Table 8:** Sources of 11 published full-text articles on 12 RCTs: systematic reviews

**No**	**Full-text articles**	**Systematic reviews**						
		
		**Evans 2001 [83]**	**Fisher 2003 [84]**	**Higgins 2003 [85]**	**Samson 2004 [9]**	**Costa 2005 [67]**	**Pham 2006 [68]**	**OHTAC 2006 [10]**
1	Braakenburg 2006 [21]	-	-	-	-	-	-	-
2	Stannard 2006 [80]*	-	-	-	-	-	-	-
3	Vuerstaek 2006 [23]	-	-	-	-	-	-	-
4	Llanos 2006 [25]	-	-	-	-	-	-	-
5	Armstrong 2005 [22]	-	-	-	-	-	-	+
6	Moisidis 2004 [18]	-	-	-	-	+	+	+
7	Moues 2004 [20]	-	-	-	+	+	+	+
8	Eginton 2003 [17]	-	-	-	+	+	+	-
9	Wanner 2003 [24]	-	-	+	+	+	+	+
10	Ford 2002 [30]	-	+	+	+	+	+	+
11	Joseph 2000 [19]	+	+	+	+	+	+	+

		**1**	**2**	**3**	**5**	**6**	**6**	**6**

* Stannard 2006 [80]: one article presenting preliminary results of 2 ongoing RCTs [40,41].

RCT: randomised controlled trial

**Table 9:** Sources of 11 published full-text articles on 12 RCTs: manufacturers, authors, congress proceedings, and authorities

		**Manufacturers**		Authors*	Congress proceedings*	Authorities*	
					
**No**	**Full-text articles**	**Kinetic Concepts, Inc.**	**Blue Sky Medical**			**Experts 2003^†^**	**Others**
1	Braakenburg 2006 [21]	+	-	-	-	-	-
2	Stannard 2006 [80]^‡^	+	-	-	-	-	-
3	Vuerstaek 2006 [23]	+	-	-	-	-	-
4	Llanos 2006 [25]	-	-	-	-	-	-
5	Armstrong 2005 [22]	+	-	-	-	-	-
6	Moisidis 2004 [18]	+	-	-	-	-	-
7	Moues 2004 [20]	+	-	-	-	-	-
8	Eginton 2003 [17]	+	-	-	-	+	-
9	Wanner 2003 [24]	+	-	-	-	+	-
10	Ford 2002 [30]	+	-	-	-	+	-
11	Joseph 2000 [19]	+	-	-	-	+	-

		**10**	**0**	**0**	**0**	**4**	**0**

* See Table 1.

† Citations submitted by experts to the German Federal Joint Committee.

‡ Stannard 2006 [80]: one article presenting preliminary results of 2 ongoing RCTs [40,41].

RCT: randomised controlled trial

### Summary of quality assessment and outcomes

#### Published trials

The overall methodological quality of the 12 published RCTs included in the IQWiG systematic review and rapid report was poor. Methodological flaws included the lack of blinding, the lack of intention-to-treat analyses, inadequate allocation concealment, the unclear definition of primary outcomes, and high drop-out rates. Significant differences in favour of NPWT for wound healing parameters, such as time to wound closure or the incidence of wound closure, were shown in 3 of 7 RCTs analysing these outcomes. Data on other patient-relevant outcomes, such as reoperation rates or pain scores, were scarce or not interpretable.

#### Unpublished trials

Because of insufficient available information on the 9 completed or discontinued unpublished RCTs, an assessment of their quality could not be conducted. Regarding the 5 discontinued industry-sponsored trials, as stated, the reasons for discontinuation were provided by KCI, which also reported the number of enrolled patients for one trial [33]. However, there was no response to a further request from IQWIG asking for more detailed information on all discontinued trials, such as the number of enrolled patients and other results data. Regarding the 4 completed RCTs with unclear sponsorship, the authors of publications did not respond to IQWiG's request to provide results data (Table 4). Consequently, the results of unpublished RCTs could not be considered in the review.

### Industrial sponsorship

The manufacturer KCI sponsored the majority (19/28) of RCTs (68% of trials referring to 85% of patients planned or analysed). The sponsorship of the remaining trials was unclear.

## Discussion

The main objective of this paper was to identify unpublished RCTs on NPWT within the framework of a systematic review. An RCT was classified as "unpublished" if no full-text paper on final study results (completed trials) or interim results (discontinued trials) was available.

An extensive search strategy was employed that included handsearching of retrievals, as well as contacting manufacturers and authors of publications. The sensitivity of bibliographic database searching for RCTs is reported to be unsatisfactory [46], and multi-source searching has been recommended to retrieve all available RCTs [47]. In this context, the usefulness of handsearching and contacting experts has been demonstrated [48,49]. The extensive search strategy was also useful to detect ongoing RCTs, of which one [22] was subsequently added to the evidence base.

A total of 28 RCTs referring to at least 2755 patients were identified. The publications on completed or discontinued RCTs reported data on less than a third of patients planned or analysed. Less than half of the conference abstracts on these trials were later published as full-text publications; similar results were found in a review by Scherer et al, who found a full-text publication rate for abstracts describing RCTs or controlled clinical trials of about 60% [50].

On the basis of results of published data alone, the IQWiG review and rapid report concluded that although there was some indication that NPWT may improve wound healing, the body of evidence available was insufficient to clearly prove an additional clinical benefit of NPWT. This finding is in line with findings from previous reviews.

Regarding the discontinued unpublished trials, according to the manufacturer the main reason for discontinuation was insufficient patient numbers. No results data for these trials were available. Regarding the completed unpublished trials, the authors of conference abstracts did not respond to IQWiG's request for information. Therefore, unpublished results data could not be considered in the IQWiG review and consequently the impact of unpublished data on the conclusions of reviews based on published data could not be assessed.

### Publication bias caused by unpublished data

Previous research has shown that inclusion of unpublished data in systematic reviews may affect the review's prior conclusions [12,13]. The most prominent example in recent years has been the evidence on serotonin reuptake inhibitors (SSRIs) in paediatric depression: a systematic review of published versus unpublished data showed that whereas published trials indicated a favourable risk-benefit profile, the addition of unpublished data reversed this profile for some SSRIs [11].

Klassen 2002 assessed the proportion of RCTs presented at a major paediatric meeting that were subsequently published as full publications; about 60% of abstracts were subsequently published, and RCTs were more likely to be published if they favoured the new therapy. The author concluded that "publication bias is a serious threat to assessing the effectiveness of interventions in child health" and called for a registry of RCTs in children so that the totality of evidence could be assessed [51].

As unpublished results data on NPWT were not available, no statement can be made on the impact of unpublished data on the validity of published evidence. However, if one assumes a "worst case scenario" (all unpublished trials show results in favour of conventional therapy), the current conclusion would no longer hold that there is some indication of a potential advantage of NPWT over conventional therapy.

Besides possible problems of statistical evaluation due to insufficient sample sizes, the reasons for the existence of unpublished RCTs on NPWT remain unclear and one can only speculate. For example, it may be possible that some of these RCTs remained unpublished because they were of poor quality or produced negative results. Previous research has shown that studies with statistically significant or positive results are more likely to be published than those with non-significant or negative results, and are also published earlier [52-54]. These studies also lead to a greater number of publications, and are more likely to be published in high impact factor journals [55]. As for discontinued trials, even if small sample sizes make statistical evaluation difficult, in our opinion the full datasets of such trials should be publicly accessible to ensure that the evidence base is complete.

The inclusion of unpublished data in systematic reviews is controversial. Although less than a third of meta-analyses include unpublished data, nearly 80% of meta-analysts and methodologists believe that unpublished data should definitely or probably be included in systematic reviews; in contrast, less than half of journal editors agree [56]. The identification of unpublished data has been recommended to minimise the risk of bias in systematic reviews [12], but there are variations in policy regarding their inclusion [57]. This may be explained by incomplete or inaccurate reporting and the subsequent difficulties in assessing the methodological quality of trials [57,58].

In general, the quality of reporting of systematic reviews on both drug and non-drug interventions is inconsistent [59]. Moreover, systematic reviews on non-drug interventions (e.g. surgical techniques) face specific problems, such as the limited quality and quantity of RCTs [60]. Searching for unpublished data and conducting sensitivity analyses to assess their impact is recommended for topics with little evidence and new or evolving interventions [58]. In their commentary on the problem of conducting systematic reviews of new health technologies, Moher and Schachter concluded that the inclusion of unpublished data was of "paramount importance" in assessing the usefulness of an intervention [61].

### The role of industrial sponsorship

Approximately two thirds of the RCTs on NPWT were sponsored by one manufacturer (KCI). Industrial sponsorship of clinical trials is common. The percentage of industry-sponsored clinical trials has increased to over 60% in recent years and the number of industry employees named as co-authors of clinical trial publications is rising [62]. However, the role of industrial sponsorship in clinical research is controversial. Industry sponsorship is discussed as being associated with the following factors: selection of inappropriate comparators, selective reporting of more favourable per protocol analyses, pro-industry conclusions, as well as restrictions on publication and data sharing [63-65]. A comparison of Cochrane reviews versus industry-supported meta-analyses showed that the latter came to more favourable conclusions on the same drugs [66]. The available data on NPWT are insufficient to make definite conclusions about the impact of industrial sponsorship in this field.

### Differences in primary study selection

Five systematic reviews on NPWT (including the IQWiG review) published in December 2004 or later showed considerable differences in the selection of primary studies published between 1994 and 2004 [6,9,10,67,68]. There was agreement between reviews regarding the selection of 4 RCTs [19,20,24,30]. One further study [17] classified by IQWiG as an eligible RCT was not classified accordingly (or identified) by 2 other reviews [10,67]. A study [69] classified as an eligible RCT by 2 reviews [9,68] was classified as non-randomised by IQWiG, and one study [70] classified as an eligible RCT by one other review [68] was also classified as non-randomised by IQWiG. These findings indicate that in systematic reviews on NPWT, search strategies, inclusion criteria, and classification of primary studies are not applied in a standardised manner.

## Conclusion

Multi-source comprehensive searches identify unpublished clinical trial data. However, lack of access to unpublished study results data raises doubts about the completeness of the evidence base on NPWT. The implementation of regulations such as prospective mandatory trial registration and the obligation to publish all results is needed to ensure that independent researchers have access to all outcomes of completed or discontinued clinical trials.

## Competing interests

The author(s) declare that they have no competing interests.

## Authors' contributions

StL initiated the study. FP coordinated the study and conducted the literature search. StL, FP, and StS screened and analysed the retrievals. NM and FP drafted the manuscript. All authors interpreted the data and made an intellectual contribution to the manuscript. All authors reviewed and approved the final version.

## Pre-publication history

The pre-publication history for this paper can be accessed here:


